# Relative Expression of PBMC MicroRNA-133a Analysis in Patients Receiving Warfarin After Mechanical Heart Valve Replacement

**Published:** 2018

**Authors:** Hamid Kabiri Rad, Mahta Mazaheri, Ali Dehghani Firozabadi

**Affiliations:** 1. International Campus, Shahid Sadoughi University of Medical Sciences, Yazd, Iran; 2. Department of Medical Genetics, Faculty of Medicine, Shahid Sadoughi University of Medical Sciences, Yazd, Iran; 3. Yazd Cardiovascular Research Center, Shahid Sadoughi University of Medical Sciences, Yazd, Iran

**Keywords:** Anticoagulation, MicroRNAs, Quantitative reverse transcription-PCR, Warfarin

## Abstract

**Background:**

MicroRNAs (miRNAs) are implicated in various biological processes including anticoagulation. However, the modulation of miRNA by pharmacological intervention such as warfarin treatment in patients receiving warfarin has not been disclosed yet. The aim of this study work was to assess the effect of warfarin drug on expression level of mir-133a-3p in patients with mechanical heart valve replacement.

**Methods:**

In this research, the expression level of miRNA-133a-3p was analyzed in Peripheral Blood Mononuclear Cells (PBMCs) from mechanical valve replacement patients who had received warfarin for at least 3 months continuously. Quantitative RT-PCR method was used for this assay.

**Results:**

Our findings indicated a significant diffrence between the rate of miR-133a-3p expression in individuals receiving warfarin and the control group (p<0.01). There was also a statistically significant difference in miR-133a-3p expression in patients with different ages (p<0.05) suggesting that the rate of miR-133a-3p expression in persons receiving warfarin is related to age. However, other variables like warfarin dose, International Normalized Ratio (INR), gender, and Body Mass Index (BMI) were not significantly effective on the miR-133a-3p experssion rate in individuals receving warfarin.

**Conclusion:**

Based on our results, it can be concluded that miR-133a-3p is involved in the coagulation pathway. The recent result indicates that warfarin affects the expression of miR-133a. This expression may be potentially important for treatment by anticoagulants. Awareness of the time course of miRNA expression profile can improve efficiency of response to warfarin.

## Introduction

The cardiovascular diseases are the most common causes of mortality worldwide. These artery diseases including coronary, cardiac ischemia, valvular failure, congenital heart defects, and heart diseases are induced by other diseases such as diabetes. Valvular disorder is one of the heart diseases which may lead to cardiac dysfunctions and, ultimately, may result in heart transplantation. One therapeutic strategy in these patients is heart valve repair and in the case of inefficacy of the repair, valve replacement including the biologic and mechanical valves should be considered. One of the most important challenges for this valve replacement is the risk of thrombosis. Patients with valve replacement of the mechanical type need to receive anti-coagulation drugs like warfarin for a lifetime due to the high risk of thrombosis ^1,2^ . The use of warfarin significantly reduces the thrombolytic damages ^3,4^ .

A vitamin K antagonist called Warfarin reduces blood clotting through disturbance in vitamin K cycle and also, it interrupts the synthesis of the coagulation factors IX, VII, II, and X through inhibiting *VKORC1*. ^5,6^ . Since the administration of warfarin is associated with a high risk of hemorrhage, its function must be repeatedly assessed *via* the laboratory test of International Normalized Ratio (INR). Increase of individuals’ hypersensitivity to warfarin consumers in different communities promotes the risk of hemorrhage and the possible subsequent death in some cases. Moreover, it has been observed that dosage adjustment basis on INR has not been successful in about 55% of cases ^7^ . The initiation of the primary dose of warfarin can be estimated based on genetic testing which leads to reduction in the time required for achieving the desirable INR. This time requirement can decrease the risk of creation of high INR (associated with haemorrhage) or low INR (associated with thrombosis). Due to genetic differences among individual patients, the prescription and administration of a specific dose of warfarin for all patients is not possible. So far, most studies have focused on factors such as genotype (genetic variant) and demographic differences (such as age, gender and weight) which may potentially affect the warfarin dosage. However, a few studies have shown the role of regulatory factors of genes involved in the metabolism of this drug ^8–10^ .

MiRNAs are a group of small single strand non-coding RNAs which interrupt the expression of genes in the post-transcription level.

The essential role of miRNAs in various biological processes such as inflammation, DNA repair, response to oxidative stress, apoptosis, cancer, and cellular growth and the post-transcription regulation of gene expression has been confirmed ^11,12^ . The inhibitory role of miR-133a in the pathogenesis of Gall Bladder Carcinoma (GBC) *via* gene targeting ^13^ and miR-133a may exert anti-tumor effects on a variety of cancers. The role of miR-133a-3p in regulating genes such as *CYP2C9* and *VKORC1* in different patients has been investigated ^14,15^ . Nonetheless, the effect of warfarin on the expression rate of miR133a-3p has not been approved. Consequently, this study tried to compare the rate of relative expression of miR-133a-3p in mechanical valve replacement patients receiving warfarin to patients in control group and also to investigate its correlation with the received warfarin dose.

## Materials and Methods

In this experimental research, the participants of this study were mechanical valve replacement patients referred to heart center of Afshar hospital in Yazd province (aged 45+ years) who had received warfarin for at least 3 months with no change in warfarin dose over the last month with an INR limit of 2–3.5. The case (12 patients) and control (10 patients) groups were matched by sex and age (Table 1).

**Table 1.** Demographic information of the patients

**Characteristics**	**Warfarin treated group (n=12)**	**Non treated group (n=10)**
**Age (years) mean±SD**	55.2±8	54.16±7.3
**Height (*cm*) mean±SD**	155.13±4.56	169.16±6.64
**Weight (*kg*) mean±SD**	69.16±6.1	68.66±5.2
**Body mass index, *kg*/*m*^2^ mean±SD**	24.27±1.59	24.1±2.6
**INR mean±SD**	2.3±0.39	
**Male/female (n/n)**	8/4	4/6
**Warfarin (mg/per week) mean±SD**	34.16±10.53	
**Aspirin, n%**	58.3	
**Statin, n%**	25	
**Hypertension, n (%)**	16.6	

Patients’ demographic information was collected by interview and through their medical records. Having obtained informed consent, all the patients voluntarily participated in the study with no treatment limitations for the patients during the completion of the study.

### Separation of peripheral blood mononuclear cells from blood samples by density gradient centrifugation

Separation of PBMCs from whole blood is usually accomplished through density gradient centrifugation using Ficoll. After the centrifugation step, Ficoll separates layers of blood, with lymphocytes and monocytes under a layer of plasma. Peripheral blood was collected in BD Vacutainer spray-coated K2EDTA Tubes. Within 2 *hr* of blood aspiration, PBMCs were isolated by using Ficoll gradient centrifugation. Buffy coat layer containing Peripheral Blood Mononuclear Cells (PBM-Cs) was stored at −80°*C* until use.

### RNA extraction and purification

Blood samples of patient were used for RNA manipulation. Firstly, for miRNA133a-3p (5′-UUUGGUCCCCUUCAACCAGCUG-3′) detection, blood samples were collected in EDTA-K2 tubes and incubated at room temperature during 1 *hr*. A two-step centrifugation (4°*C* at 820×*g* for 10 *min*, then 4°*C* at 16000×*g* for 10 *min*) was done and then the supernatant phase was transferred to RNase/DNase-free tubes and stored at −80°*C*^15^. Total RNA was extracted by plasma samples using the column RNA isolation Kit (Denazist, Iran) according to the manufacturer’s instructions. The purity and concentration of RNA were determined by OD 260/280 readings using a Nanodrop spectrophotometer (Nanodrop, Thermo Scientific, Germany). RNA integrity was determined by capillary electrophoresis (Biorad, Germany). The quantity and quality of RNA was assayed by using nanodrop spectrophotometer (Nanodrop, Thermo Scientific, Germany) and 2% agarose gel electrophoresis, respectively. In the case of the presence of two 18S and 28S ribosomal RNA bands, the quality of the extracted RNA was approved. The analysis of miRNA-133a expression in PBMCs was performed by miRNA synthesis kit using the Universal RT miRNA PCR, Polyadenylation and cDNA synthesis kit (Pars Genome Co., Iran). cDNA was diluted 5× and assayed in 20 *μl* PCRs according to the protocol for RT miRNA PCR.

### Quantitative real time PCR analysis

Expression analysis of miRNA-133a was carried out in an Applied Biosystem Step One plus Real-Time PCR System (ABI, USA). PCR MasterMix for Syber Green Assays (Hot Tag EvaGreen, ROX, GeneAll, South Korea) was used to monitor light cycles of PCR, according to the manufacturer’s protocol. The amplification condition was 95°*C* for 5 *min*, 40 cycles at 95°*C* for 15 *s* and 60°*C* for 1 *min*. Melting curves analysis was performed after each reaction to exclude non specific amplifications. The optimal baseline and threshold values were determined using automatic CT function available. Relative gene expression levels for the three replications were calculated using the 2 ^−**ΔCT**^ method ^16^ . Melting curve was used to determine the specific amplification of the specific gene segments.

### Data analysis

The measurement of the cDNA samples was performed in duplicate. After obtaining the pair CT for each sample, their means were calculated. By importing the data to SPSS19, the outliers were first identified and excluded from the study along with their control samples. The results were measured as mean±SD. T-test and One-way ANOVA were used to compare the means between the two groups. Each test was repeated at least twice and performed independently.

## Results

**Figure 1.**
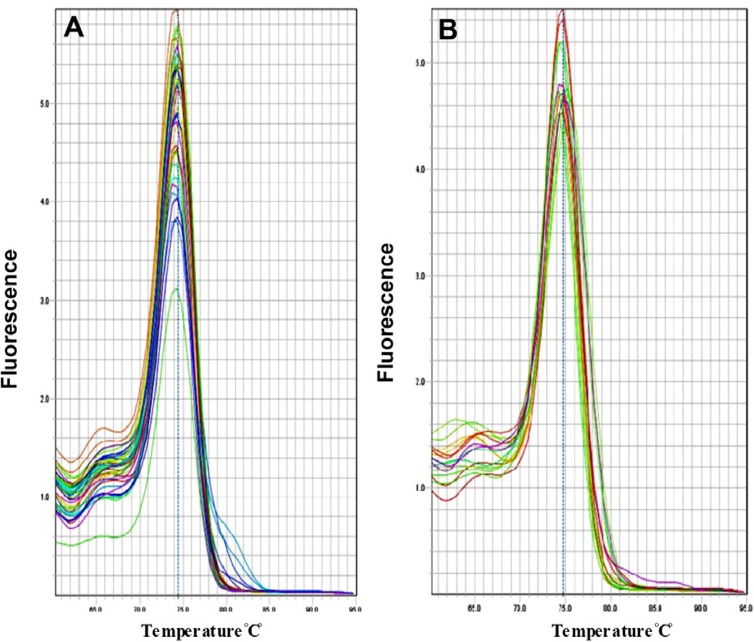
A) The melting curve of snRNA-U6 and B) miR-133a-3p A on the basis of temperature (horizontal axis) and diffenential fluerescent signal (vertical axis) received from the device.

**Figure 2.**
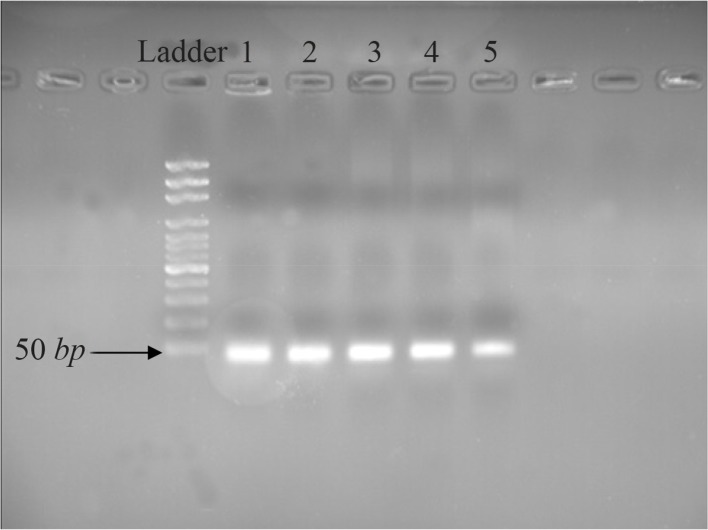
Different concentrations of RT-PCR pruduct of 2% agarose electrophoresis agarose. Line 1: 4000 *ng/μl*; Line 2: 2000 *ng/μl*; Line 3: 1000 *ng/μl*; Line 4: 700 *ng/μl* and Line 5: 350 *ng/μl.*

The fluorescent light was continuously monitored and the temperature was gradually increased from 60 to 95°*C* by the device. The melting curve of miR-133a-3p and snRNA-U6 was obtained as a single peak curve indicating the proliferation of the intended specific product and lack of its genomic DNA contamination (Figures 1A and 1B). Furthermore, the qPCR product was placed on 2% Agarose Gel. A molecular 50 *bp* DNA Ladder was used in the reaction above and fragments shorter than 50 *base pairs* were considered as specific targets. There was only one specific band (approximately 48 *bp*) in each of the reactions done with primers miR-133a-3p and snRNA-U6 confirming the specificity of PCR products in our sample (Figure 2).

**Figure 3.**
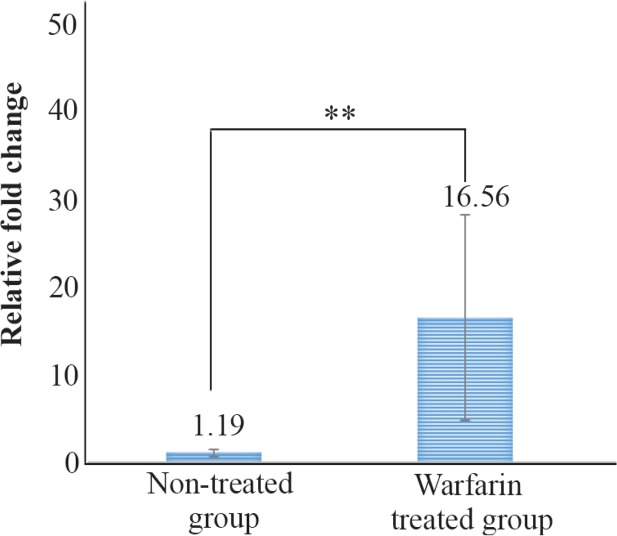
Comparison of means of miR-133a-3p expression rate between warfarin treated group and non-treated group. The difference was statistically significant ** (p<0.01).

A comparison of the mean of miR-133a-3p expression in patients receving warfarin and the patients in control group demonstrated that the expression levels were higher in warfarin receivers indicating a significant differnce between the two groups (p<0.01) (Figure 3).

**Figure 4.**
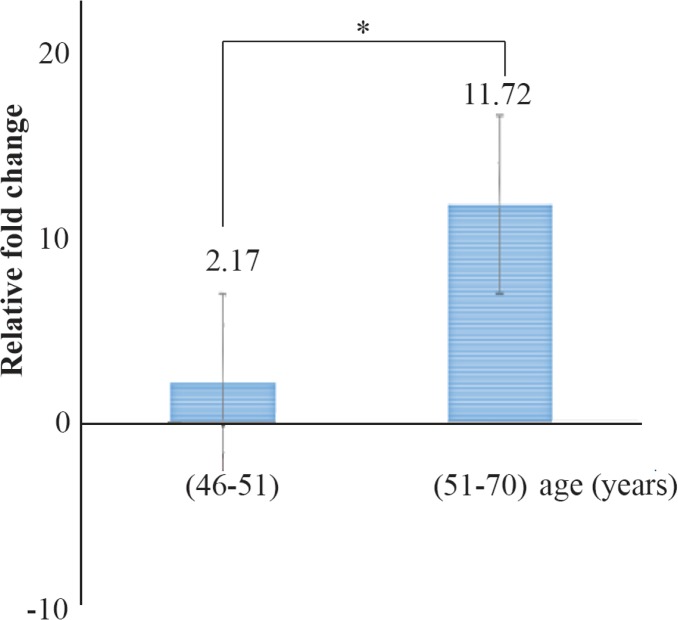
Comparison of means miR-133a expression level rate between two different age groups receiving warfarin. The difference was statistically significant (p<0.05).

A comparison of mean of miR-133a-3p expression rate between two different age groups receiving warfarin demonstrated that the expression levels were higher in age group (51–70) indicating a significant difference between the two groups (p<0.05) (Figure 4).

Other variables like warfarin dose, gender, INR and BMI were not significantly effective on the miR-133a-3p experssion rate in individuals receving warfarin.

## Discussion

Due to individual differences, the patients’ response to warfarin is different. Some studies have indicated that at least 30% of the difference in dosage is attributed to genetic variants in *CYP2C9* and *VKORC1* genes ^15^ . Also, 1–7% of dose changes required for warfarin is related to *CYP4F2* gene ^17^ . On the whole, 60% of the changes is attributable to a combination of various factors such as height, weight, and the genetic variants including *VKORC1*, *CYP4F2*, and *CYP2C9*. In any case, about 30–40% of drug dosage changes are not known yet ^18^ .

It has been observed that diseases and different compounds affect the expression of miRNAs. Expression of multiple miRNAs in the circulation of the plaque for Unstable Angina (UA) patients showed the important roles of this drug on regulating signal path-ways for the pathogenesis of UA ^19^ . The expression levels of miR-22 and miR-133a-3p significantly increased and decreased, respectively by induction of cardiomyocyte hypertrophy by using angiotensin II (hypertrophy inducer). Nonetheless, after treatment of cardiomyocytes with atorvastatin, the expression level of miR-22 decreased significantly, but the expression level of miR-133a-3p did not change significantly ^20^ which is in agreement with our results. The physical features of individuals (age, gender, and race) affect the expression of both mRNA and miRNA. It has been observed that the mRNA and miRNA content of platelet varies with respect to gender. Simon *et al* showed in their study that miR-223 is one of the nine miRNAs that are different in men and women. Interestingly, of seven mRNAs which vary in the two genders, three were the potential targets of miR-223 ^21^ .

In this study, different samples derived from different age group were used. Nevertheless, there was no significant difference between the groups of men and women who received warfarin in our study which could be caused by the nature and function of drudge. In this work, any correlation between miR-133a-3p expression and BMI was observed which is in agreement with similar works ^21,22^ . There was a significant difference in miRNA expression level in our study between the two different age groups receiving warfarin. Regarding the profile expression of miRNAs related to age in regulating the expression of genes involved in drug metabolism, the results of two studies indicated that the expression of miRs-128, -106a, -18a, -484, 548a-3p, and 425 decreased significantly in both platelet and PBMC ^21,23^ . It seems that a wide range of physiologic pathways contribute to the age and life span of individuals and it is believed that the genetics of age is complex. Normalization of miRNA expression with internal control is of utmost importance. Some studies show that the simultaneous use of several internal controls for exactly measuring the changes in expression is more suitable ^24,25^ . Unfortunately, snRNA-U6 did not manifest a stable expression in our study. Regarding the role of *VKORC1* gene in the coagulation pathway and prevention of vascular calcification, it seems that warfarin has some effect on the expression of miR-133a.

Furthermore, some studies have indicated that warfarin exerts an anticarcinogenic and anti-inflammatory effect through inhibiting proteins of the signaling pathway (mitogen-activated protein kinase (MAPK/ERK) that regulate various activities of this pathway including cell proliferation, mitosis, cell motility, metabolism, and apoptosis ^26^ . It has been observed that warfarin in low concentrations has anti-inflammatory effects due to inhibition of secretion of Il-6 and the messaging path Tyro3/Gas6 while it is pre-inflammatory in high concentrations ^27,28^ . It seems that miRNAs contribute to the regulation of genes involved in ADME of drugs under physiologic conditions like inflammation. Additionally, the regulation of the expression of other genes involved in warfarin metabolism like *CYP2C9* gene by miR-130b related to inflammation has been indicated ^29^ . Considering the controlling role of miR-133a-3p in the coagulation pathway, the recent result indicates that warfarin affects the expression of miR-133a. This expression may be potentially important for treatment by anticoagulants.

## Conclusion

Awareness of miRNA expression patterns is important in identifying preventing and therapeutic strategies in patients receiving warfarin after mechanical heart valve replacement that are at risk of thrombosis and in selection of suitable dose of warfarin as a prognosis and diagnosis marker. An awareness of the time course of miRNA expression profile can improve efficiency of response to warfarin. Type of response to drug can help to effective treatment by an awareness of the course of expression of miRNAs. These results show that miRNAs as molecular interference regulators are involved in regulation and modulation of several genes implicated in homeostatis and hemostasis in normal, pathogenic and pharmaceutical conditions, so they influence gene expression of target genes.

## References

[1] Cannegieter SC Rosendaal F Wintzen A Van der Meer FJ Vandenbroucke JP Briët E Optimal oral anticoagulant therapy in patients with mechanical heart valves. N Engl J Med 1995;333(1):11–17. 7776988 10.1056/NEJM199507063330103

[2] Li Y Zhu J Ding JQ VKORC1 rs2359612 and rs-9923231 polymorphisms correlate with high risks of cardiovascular and cerebrovascular diseases. Genet Mol Res 2015;14(4):14731–14744. 26600534 10.4238/2015.November.18.38

[3] [No authors listed] Effect of long-term oral anticoagulant treatment on mortality and cardiovascular morbidity after myocardial infarction. Anticoagulants in the secondary prevention of events in coronary thrombosis (ASPECT) research group. Lancet 1994;343(8896):499–503. 7906757

[4] Drapkin A Merskey C Anticoagulant therapy after acute myocardial infarction. Relation of therapeutic benefit to patient’s age, sex, and severity of infarction. JAMA 1972; 222(5):541–548. 4117261 10.1001/jama.222.5.541

[5] Oldenburg J Watzka M Rost S Müller C VKORC1: molecular target of coumarins. J Thromb Haemost 2007; 5 Suppl 1:1–6. 10.1111/j.1538-7836.2007.02549.x 17635701

[6] Sabir I Khavandi K Brownrigg J Camm AJ Oral anticoagulants for asian patients with atrial fibrillation. Nat Rev Cardiol 2014;11(5):290–303. 24614113 10.1038/nrcardio.2014.22

[7] Lane DA Lip GY Maintaining therapeutic anticoagulation: the importance of keeping “within range”. Chest 2007;131(5):1277–1279. 17494777 10.1378/chest.07-0273

[8] Sconce EA Kamali F Appraisal of current vitamin K dosing algorithms for the reversal of over anticoagulation with warfarin: the need for a more tailored dosing regimen. Eur J Heamathol 2006;77(6):457–462. 10.1111/j.0902-4441.2006.t01-1-EJH2957.x 17042764

[9] International Warfarin Pharmacogenetics Consortium Klein TE Altman RB Eriksson N Gage BF Kimmel SE Estimation of the warfarin dose with clinical and pharmacogenetic data. N Engl J Med 2009;360(8): 753–764. 19228618 10.1056/NEJMoa0809329 PMC2722908

[10] Pérez-Andreu V Roldán V Antón AI García-Barberá N Corral J Vicente V Pharmacogenetic relevance of CYP4F2 V433M polymorphism on acenocoumarol therapy. Blood 2009;113(20):4977–4979. 19270263 10.1182/blood-2008-09-176222

[11] Esteller M Non-coding RNAs in human disease. Nat Rev Genet 2011;12(12):861–874. 22094949 10.1038/nrg3074

[12] Almeida MI Reis RM Calin GA MicroRNA history: Discovery, recent applications, and next frontiers. Mutat Res 2011;717(1–2):1–8. 21458467 10.1016/j.mrfmmm.2011.03.009

[13] Huang Y Wu Y Dong J Han D Yang S Lin J Micro-RNA-133a-3p exerts inhibitory effects on gallbladder carcinoma via targeting RBPJ. Am J Cancer Res 2016;6 (11):2448–2462. 27904763 PMC5126265

[14] Wang GK Zhu JQ Zhang JT Li Q Li Y He J Circulating microRNA: a novel potential biomarker for early diagnosis of acute myocardial infarction in humans. Eur Heart J 2010;31(6):659–666. 20159880 10.1093/eurheartj/ehq013

[15] Flockhart DA O’Kane D Williams MS Watson MS Flockhart DA Gage B Pharmacogenetic testing of CYP2C9 and VKORC1 alleles for warfarin. Genet Med 2008;10(2):139–150. 18281922 10.1097/GIM.0b013e318163c35f

[16] Pfaffl MW A new mathematical model for relative quantification in real-time RT-PCR. Nucl Acid Res 2001;29(9):e45. 10.1093/nar/29.9.e45 PMC55695 11328886

[17] Borgiani P Ciccacci C Forte V Sirianni E Novelli L Bramanti P CYP4F2 genetic variant (rs2108622) significantly contributes to warfarin dosing variability in the Italian population. Pharmacogenomics 2009;10(2): 261–266. 19207028 10.2217/14622416.10.2.261

[18] Sconce EA Khan TI Wynne HA Avery P Monkhouse L King BP The impact of CYP2C9 and VKORC1 genetic polymorphism and patient characteristics upon warfarin dose requirements: proposal for a new dosing regimen. Blood 2005;106(7):2329–2333. 15947090 10.1182/blood-2005-03-1108

[19] Li J Chen H Ren J Song J Zhang F Zhang J Effects of statin on circulating microRNAome and predic ted function regulatory network in patients with unstable angina. BMC Med Genomics 2015;8:12. 25889164 10.1186/s12920-015-0082-4 PMC4364658

[20] Tu Y Wan L Bu L Zhao D Dong D Huang T MicroRNA-22 downregulation by atorvastatin in a mouse model of cardiac hypertrophy: a new mechanism for antihypertrophic intervention. Cell Physiol Biochem 2013;31(6):997–1008. 23860036 10.1159/000350117

[21] Simon LM Edelstein LC Nagalla S Woodley AB Chen ES Kong X Human platelet microRNA-mRNA networks associated with age and gender revealed by integrated plateletomics. Blood 2014;123(16):e37–45 24523238 10.1182/blood-2013-12-544692 PMC3990915

[22] Okuhara A Nakasa T Shibuya H Niimoto T Adachi N Deie M Changes in microRNA expression in peripheral mononuclear cells according to the progression of osteoarthritis. Mod Rheumatol 2012;22(3):446–457. 22006119 10.1007/s10165-011-0536-2

[23] Noren Hooten N Abdelmohsen K Gorospe M Ejiogu N Zonderman AB Evans MK microRNA expression patterns reveal differential expression of target genes with age. PLoS One 2010;5(5):e10724. 20505758 10.1371/journal.pone.0010724 PMC2873959

[24] McDermott AM Kerin MJ Miller N Identification and validation of miRNAs as endogenous controls for RQ-PCR in blood specimens for breast cancer studies. PLoS One 2013;8(12):e83718. 24391813 10.1371/journal.pone.0083718 PMC3877087

[25] Peltier HJ Latham GJ Normalization of microRNA expression levels in quantitative RT-PCR assays: identification of suitablereference RNA targets in normal and cancerous human solid tissues. RNA 2008;14(5):844–852. 18375788 10.1261/rna.939908 PMC2327352

[26] Han S Zhou V Pan S Liu Y Hornsby M McMullan D Identification of coumarin derivatives as a novel class of allosteric MEK1 inhibitors. Bioorg Med Chem Lett 2005;15(24):5467–5473. 16199156 10.1016/j.bmcl.2005.08.097

[27] Kater AP Peppelenbosch MP Brandjes DP Lumbantobing M Dichotomal effect of the coumadin derivative warfarin on inflammatory signal transduction. Clin Diagn Lab Immunol 2002;9(6):1396–1397. 12414784 10.1128/CDLI.9.6.1396-1397.2002 PMC130100

[28] Kurohara M Yasuda H Moriyama H Nakayama N Sakata M Yamada K Low-dose warfarin functions as an immunomodulator to prevent cyclophosphamide-induced NOD diabetes. Kobe J Med Sci 2008;54(1):E1–E13. 18772604

[29] Rieger JK Reutter S Hofmann U Schwab M Zanger UM Inflammation-associated microRNA-130b down-regulates cytochrome P450 activities and directly targets CYP2C9. Drug Metab Dispos 2015;43(6):884–888 25802328 10.1124/dmd.114.062844

